# Hybrid CNN-Transformer Model for Accurate Impacted Tooth Detection in Panoramic Radiographs

**DOI:** 10.3390/diagnostics15030244

**Published:** 2025-01-22

**Authors:** Deniz Bora Küçük, Andaç Imak, Salih Taha Alperen Özçelik, Adalet Çelebi, Muammer Türkoğlu, Abdulkadir Sengur, Deepika Koundal

**Affiliations:** 1 Department of Software Engineering, Faculty of Engineering, Samsun University, 55000 Samsun, Turkey; 230708002@samsun.edu.tr (D.B.K.); muammer.turkoglu@samsun.edu.tr (M.T.); 2 Department of Electrical and Electronic Engineering, Faculty of Engineering, Munzur University, 62000 Tunceli, Turkey; andacimak@munzur.edu.tr; 3 Department of Electrical and Electronic Engineering, Faculty of Engineering, Bingol University, 12000 Bingol, Turkey; 4 Oral and Maxillofacial Surgery Department, Faculty of Dentistry, Mersin University, 33000 Mersin, Turkey; adalet_celebi@hotmail.com; 5 Department of Electrical and Electronic Engineering, Faculty of Technology, Firat University, 23100 Elazig, Turkey; ksengur@firat.edu.tr; 6 A.I. Virtanen Institute for Molecular Sciences, Faculty of Health Sciences, University of Eastern Finland, 70211 Kuopio, Finland; dkoundal@uef.fi

**Keywords:** impacted tooth detection, YOLO, transformer, super resolution, Weighted Boxes Fusion

## Abstract

**Background/Objectives:** The integration of digital imaging technologies in dentistry has revolutionized diagnostic and treatment practices, with panoramic radiographs playing a crucial role in detecting impacted teeth. Manual interpretation of these images is time consuming and error prone, highlighting the need for automated, accurate solutions. This study proposes an artificial intelligence (AI)-based model for detecting impacted teeth in panoramic radiographs, aiming to enhance accuracy and reliability. **Methods:** The proposed model combines YOLO (You Only Look Once) and RT-DETR (Real-Time Detection Transformer) models to leverage their strengths in real-time object detection and learning long-range dependencies, respectively. The integration is further optimized with the Weighted Boxes Fusion (WBF) algorithm, where WBF parameters are tuned using Bayesian optimization. A dataset of 407 labeled panoramic radiographs was used to evaluate the model’s performance. **Results:** The model achieved a mean average precision (mAP) of 98.3% and an F1 score of 96%, significantly outperforming individual models and other combinations. The results were expressed through key performance metrics, such as mAP and F1 scores, which highlight the model’s balance between precision and recall. Visual and numerical analyses demonstrated superior performance, with enhanced sensitivity and minimized false positive rates. **Conclusions:** This study presents a scalable and reliable AI-based solution for detecting impacted teeth in panoramic radiographs, offering substantial improvements in diagnostic accuracy and efficiency. The proposed model has potential for widespread application in clinical dentistry, reducing manual workload and error rates. Future research will focus on expanding the dataset and further refining the model’s generalizability.

## 1. Introduction

Impacted teeth are generally defined as situations in which the tooth cannot take its place in the mouth even though it is time to erupt, and based on clinical and radiologic evaluations, it is not possible to erupt through the natural process. Teeth may remain impacted in bone or soft tissue [1,2]. Teeth may remain impacted due to local and systemic factors. Local factors include deciduous tooth retention, obstacles in the eruption pathway, insufficient arch length, malposed tooth germs, and cleft lip and palate, while systemic factors include cleidocranial dysplasia, febrile diseases, Down syndrome, and hormonal irregularities. Mandibular third molars have one of the highest rates of impacted teeth among all teeth. They are followed by maxillary third molars, maxillary canines, and mandibular premolars [2].

Today, with the integration of artificial intelligence models in dentistry, as in any other field, there are promising results in the detection of dental conditions other than caries and calculus on panoramic radiographs. Thanks to the development of artificial intelligence models in all areas of dental radiology, it has been observed that they assist the clinician in panoramic diagnosis and treatment planning, as well as digitally based student education, especially during the pandemic period [3]. Artificial intelligence models also facilitate the detection of impacted third molars. In this regard, early diagnosis for the clinician and early treatment for the patient will be convenient. Artificial intelligence applications show high accuracy in detecting impacted third molars and their relationship with anatomical structures [4].

With the development of technology, revolutionary changes have taken place in the field of radiology from the past to the present. Studies on the development of patient care through artificial intelligence-based systems in radiographic images in the field of oral and dental health continue. With a growing population and a proportional increase in the number of patients, the inspection of panoramic dental images is a tiring and time-consuming process for experts. In addition, situations may be missed due to clinicians’ lack of experience [5,6]. To address this problem, research into the integration of computer-aided diagnostic tools in oral radiology, as in many other areas of medical imaging, is ongoing. In this way, artificial intelligence-based diagnostic systems provide an important solution to reduce the workload of experts by assisting in the evaluation of clinical data. Oral health plays an important role in a person’s overall health and quality of life. In addition, early detection of dental and oral health problems is important as they also cause systemic diseases in the body, such as respiratory infections [7]. Many applications of artificial intelligence-based oral radiology to oral and dental health have been proposed in the literature. These have mainly focused on areas such as the pathology of oral lesions [8,9], classification of teeth, materials, and dental implants [10,11,12,13,14,15], detection of periodontally problematic teeth [16], periodontitis [17,18,19,20], maxillary sinus [21,22,23,24,25,26,27,28,29,30], and dental caries [31,32,33,34,35,36,37,38,39,40,41,42]. Few studies have been conducted on impacted teeth in the literature compared to the detection of caries, maxillary sinus, and periodontal problems. Orhan et al. introduced a Convolutional Neural Network-based system for the detection of impacted third molars in cone beam computed tomography (CBCT) images. In their experimental study, they achieved a success rate of 86.2% in detecting impacted teeth [4]. Kuwada et al. used three deep learning architectures, AlexNet, VGG-16, and DetectNet, to detect maxillary sinus lesions and impacted third molars. In their experimental studies with three different deep architectures, they achieved a maximum accuracy of 96% in detecting impacted teeth with the DetectNet architecture [41]. Durmuş et al. proposed a deep architecture based on a ResNet backbone network for impacted tooth detection. They achieved 85.14% intersection over union (*IoU*) performance with the ResNet18 backbone network in the Pyramid Scene Parsing Network (PSPNet) architecture they developed [43]. Başaran et al. used the R-CNN Inception v2 model to evaluate ten different tooth conditions. The deep architecture they used on panoramic images achieved an F1 score performance of 86.25% [3]. Zhicheng et al. developed a SAM model for the detection of impacted teeth. They achieved an accuracy value of 86.73% with their proposed method [5]. Imak et al. proposed an improved version of U-Net called ResMIBCU-Net for the detection of impacted teeth. An accuracy value of 99.82% was observed in the proposed pixel-based method [44]. Celik investigated the effects of two detectors, Faster R-CNN and YOLOv3, on the detection of impacted third molars. In his experimental studies, Faster R-CNN achieved better performance than the YOLOv3 detector when compared to the results obtained with different backbone networks. A mean accuracy performance (mAP) of 96% was achieved with the proposed YOLOv3 detector [45]. In the current literature on impacted teeth, although the results are informative, the success results obtained with a limited number of applications are not at a sufficient level. More research is needed, and ongoing studies combining different innovative methods are required.

In this research, an artificial intelligence-based real-time learning framework is proposed for the detection of affected teeth in dentistry. The proposed model uses super-resolution methods to improve the resolution of panoramic radiography images. These methods are techniques used to convert low-resolution images into high-resolution images. In this way, the details in the image are improved, and the performance of the model is enhanced with clearer and sharper images.

The main contributions of this study are as follows:

- Integration of deep learning algorithms, including super-resolution techniques, CNNs, transformer-based models, and the Weighted Boxes Fusion (WBF) component, to improve the accuracy and efficiency of object detection in panoramic radiography images.
- Optimization of the model for accurate and real-time detection of impacted teeth in panoramic radiographs, assisting dentists in their clinical decision-making processes and contributing to more effective patient care.
- High detection accuracy validated through experimental studies, demonstrating that the model can reliably detect impacted teeth in panoramic images.
- Highlighting the potential of AI-based tools in dentistry to automate manual analysis processes, reduce the workload of experts, and minimize human error. These contributions show that the proposed model can have a wide range of applications in both academic and clinical settings, establishing a future reference point for artificial intelligence-based solutions in dentistry.

## 2. Materials and Methods

In this study, a multi-component deep learning model was developed to enhance the analysis of panoramic radiography images. The proposed model utilizes super-resolution techniques to convert low-resolution images into high-resolution representations, enhancing image clarity and detail. This improvement facilitates better performance in diagnostic applications.

The model integrates Convolutional Neural Networks (CNNs) and transformer architectures for regional object detection. CNNs are employed to identify and classify features in the images, while transformers leverage attention mechanisms to model relationships in sequential data. Additionally, a Weighted Boxes Fusion (WBF) component is incorporated to combine the outputs of multiple object detection models, ensuring more accurate and reliable predictions.

**Figure 1:**
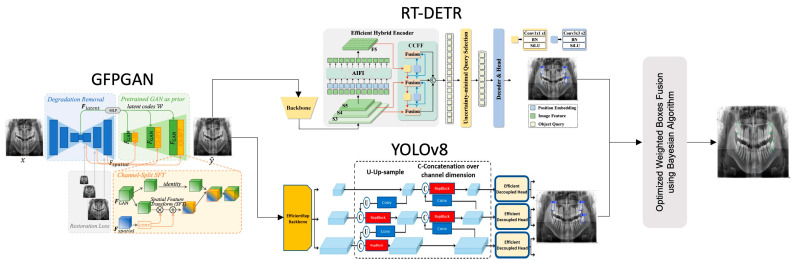
Schema of the proposed architecture.

The parameters of the model were rigorously optimized through iterative trials and fine-tuning to maximize performance. These adjustments were designed to achieve the best fit for the training data and to ensure the model operates efficiently. A general representation of the proposed model is provided in Figure 1. In this research, an artificial intelligence-based real-time learning framework is proposed for the detection of affected teeth in dentistry. The proposed model is based on the combination of Convolutional Neural Networks (CNNs) and transformer models based on super-resolution images. The basic architectures that make up the proposed model are further explained in more detail in the following subsections.

The proposed model, shown in Figure 1, consists of four main components. Each component provides an optimized function for the detection of dental structures in panoramic images:

- The Generative Facial Prior (GFP-GAN) module improves the clarity of the input image by enhancing low-resolution panoramic images with a super-resolution method. This process provides higher quality data for the next steps and contributes significantly to the overall performance of the model. GFP-GAN reconstructs the detail in low-resolution images, producing a sharper and more meaningful input.
- In the second phase, the RT-DETR module uses a transformer-based approach to regionally detect dental structures in panoramic images. This module extracts meaningful features from complex image structures and accurately labels target regions. In particular, it provides high accuracy in dental radiographs thanks to its ability to model sequential relationships and improve positional accuracy.
- In the third stage, the YOLOv8 model classifies the detected tooth regions in detail and marks them more precisely. YOLOv8 is one of the most efficient object detection algorithms, delivering fast and accurate results. This component allows detailed analysis of important structures in dental radiographs and optimizes the detected areas, improving overall accuracy.
- In the final stage, the detection results from the different modules are combined with the WBF method, enhanced with Bayesian optimization. This method combines the strengths of the models and provides a more accurate and integrated output. By combining the predictions of different modules, WBF minimizes the false positive and false negative rates. Finally, the analysis of the dental structures is complete, and the results are presented with high accuracy.

The algorithms and architectures used in these process steps are described in more detail in the subheadings. The function and benefits of each component contribute significantly to the overall success of the model. This integrated structure provides an innovative solution for high accuracy, precision, and reliability in dental radiography.

### 2.1. Generative Facial Prior

Generative Facial Prior (GFP-GAN), proposed by Wang et al., (2021), is an advanced GAN (Generative Adversarial Network)-based model for producing high-resolution images from low-resolution inputs [46]. Image reconstruction generally includes a structure consisting of U-Net-based encoder and decoder parts. Specifically, StyleGAN2 [47] stands out as a technology with strong production capability when used as a front module. A pre-trained StyleGAN2 module provides a robust ability to restore detailed regions in the image by utilizing the rich features of the semantic information extracted from the encoder. The degradation removal module increases the expressive power of the model by retaining semantic attributes and extracting deep feature information during the convolution process, as shown in Equation (1) and Equation (2), respectively [46]:

(1) $$W=MLP(F_{latent})$$

(2) $$F_{GAN}=StyleGAN2(W)$$

Here, the $F_{latent}$ coding vector, generated by the degradation removal process, encodes meaningful features from the input image. It is then processed through Multi-Layer Perceptron (MLP) layers to produce the latent code W, which is passed into StyleGAN2 to extract deep, meaningful features. These steps allow the model to focus on fine-grained details necessary for high-quality restoration. In order to remove blurs and enhance the final restoration quality, the model uses a comprehensive set of loss functions. Reconstruction Loss (L_rec_) ensures the restored image closely resembles the ground truth, while Adversarial Loss (L_adv_) enhances the production of realistic textures. Additionally, Facial Component Loss (L_comp_) improves key facial areas such as the eyes and mouth, and Identity Preserving Loss (L_id_) maintains the identity of the restored face. This combination of losses enables better compatibility between the encoder and decoder, improving both realism and fidelity [46,47,48,49]. The comprehensive training process, supported by these loss functions, allows GFP-GAN to excel not only in face restoration but also in applications like panoramic dental imaging. For example, it effectively enhances details such as fractures, cavities, or impacted teeth, demonstrating its versatility and practical potential in medical imaging and other image enhancement tasks.

### 2.2. CNN (YOLO)

The YOLO (You Only Look Once) series, which offers fast and accurate solutions in the field of object detection, continues to develop constantly. One of these developments is the YOLOv8 model. YOLOv8 stands out as an important step forward in the field of computer vision.

- Main Network: YOLOv8 uses a modified version of CSPDarknet53 as its main network. CSPDarknet53 is replaced by the C2f Module, which uses gradient shunt connectivity to enrich the information flow and maintains a lightweight structure. Furthermore, the GIS Module performs processing using convolution, group normalization, and SiLU activation. YOLOv8 also uses the SPPF Module to convert the input feature maps into a fixed-size map, which reduces computational effort and lowers latency.
- Neck: The neck structure of YOLOv8 uses the PAN-FPN structure. Inspired by PANet, this structure improves location information by combining features at different scales and provides feature diversity and completion.
- Head: YOLOv8 uses two separate branches for segmentation and bounding box regression. For classification purposes, binary cross-entropy loss (BCE Loss) is applied, whereas distribution focal loss (DFL) and CIoU are utilized for tasks involving bounding box regression. The model also uses a non-anchor detection model and improves detection accuracy and robustness by assigning by task.

With these components, YOLOv8 provides improved detection accuracy and speed over previous models in the YOLO series. With its modular structure and innovative components, it sets a high standard in the field of computer vision [50,51,52].

### 2.3. Transformer (RT-DETR)

The RT-DETR (Real-Time Detection Transformer) model, developed by Baidu, offers an innovative approach to object detection by combining high accuracy and real-time performance [53]. The model includes components that provide significant improvements in both speed and accuracy. These are as follows [53,54]:

1. Backbone Network: RT-DETR uses convolution-based networks such as ResNet or HGNetv2 as the backbone. The last three stages of the backbone (S3, S4, S5) serve as the input for the hybrid encoder and enable efficient processing of multi-scale features. This design allows the model to be built on a strong foundation and provides a suitable structure for modeling multi-scale information.
2. Neck: The neck structure of RT-DETR is not designed as a distinct intermediate layer as in conventional models but instead offers an innovative structure integrated within the Hybrid Encoder. The Neck function is realized by the following two modules:
  - Attention in Feature Interaction (AIFI): This module is specifically designed to improve semantic information in deep feature maps (S5). AIFI enhances information flow by modeling dependencies between features. This approach provides high performance with a more minimal design unlike classical neck structures.
  - Cross-Scale Feature-Fusion Module (CCFM): Combining features at different scales (S4 and S5) enables the model to effectively use multi-scale information. Instead of a classical feature pyramid, CCFM offers a lighter and more flexible fusion mechanism.

This innovative approach allows the model to remain lightweight while avoiding the additional computational burden seen in traditional neck structures. On the other hand, the head part of this architecture is as follows [53,54]:

3. Head: Unlike conventional object detection models, RT-DETR’s head structure is designed to be more dynamic and integrated with the encoder. This structure works as follows:
  - Decoder: The IoU-aware Query Selection mechanism selects the most relevant object queries from the features received from the encoder and optimizes them to generate class information and bounding boxes. This mechanism reduces redundant computations and improves accuracy by focusing on the most important objects in the scene.
  - Auxiliary Prediction Heads: These heads enable the model to learn faster and more accurately by making intermediate predictions. Unlike the separate classification and regression heads in classical models, it works while integrated into the decoder and does not require an extra ‘head’ layer.

RT-DETR provides a lighter, faster, and scalable solution by offering a hybrid and integrated design instead of the distinct neck and head structures in classical models. This innovative approach reduces the computational burden and is optimized for real-time performance. In this way, RT-DETR represents a significant paradigm shift in the field of object detection [53,54].

### 2.4. Ensemble Strategy

In this study, an ensemble strategy was used to combine the strengths of the YOLO and RT-DETR models. Ensemble methods aim to improve accuracy, precision, and reliability by combining the predictions of different models. In particular, the fast real-time detection capabilities of YOLO and the long-range correlation learning capability of RT-DETR are combined to achieve superior performance in the detection of impacted teeth. The ensemble strategy used in this study is based on the Weighted Boxes Fusion (WBF) algorithm [54]. The WBF algorithm assigns a weight to each box when fusing bounding box predictions from multiple models. This process optimizes the contribution of each bounding box to the model prediction, resulting in a more accurate result.

The steps of the WBF algorithm are as follows [55,56,57,58]:

- The combined confidence score C of the fused bounding box is calculated as the average of the confidence scores $C_{i}$ of all participating bounding boxes:

(3) $$C=\frac{\sum_{i=1}^{T}C_{i}}{T}$$

Here, T represents the total number of bounding boxes contributing to the fusion.

- The x,y,w,h coordinates of the fused bounding box are computed as weighted averages of the corresponding coordinates of the individual bounding boxes:

(4) $$x=\frac{\sum_{i=1}^{T}C_{i}\cdot x_{i}}{\sum_{i=1}^{T}C_{i}}$$

(5) $$y=\frac{\sum_{i=1}^{T}C_{i}\cdot y_{i}}{\sum_{i=1}^{T}C_{i}}$$

(6) $$w=\frac{\sum_{i=1}^{T}C_{i}\cdot w_{i}}{\sum_{i=1}^{T}C_{i}}$$

(7) $$h=\frac{\sum_{i=1}^{T}C_{i}\cdot h_{i}}{\sum_{i=1}^{T}C_{i}}$$

Here, $C_{i}$ is the confidence score of the i−th bounding box, and $x_{i},y_{i},w_{i},h_{i}$ represent the center coordinates, width, and height of the i−th bounding box.

(8) $$IoU=\frac{Area\;of\;Overlap}{Area\;of\;Union}$$

Bounding boxes with *IoU* values below the threshold are excluded from the results. This step ensures that the fused box retains only the most relevant predictions, eliminating excessive overlap.

In summary, Weighted Boxes Fusion (WBF) is an efficient method that combines bounding boxes estimated by different object detection models by optimizing them according to their confidence values and model weights. This approach aims to emphasize the strengths of the models while minimizing their weaknesses, thereby significantly improving the accuracy, consistency, and reliability of the combined predictions [55,56,57]. In this study, the strengths of the YOLO and RT-DETR models are combined using the WBF algorithm, and a high-performance solution for impacted tooth detection is presented.

### 2.5. Optimization

In this study, Bayesian optimization is used to determine the optimal parameters of the developed model and to improve the detection performance [58]. Unlike traditional grid search and random search methods, Bayesian optimization makes hyperparameter search more efficient and continuously improves model performance in an iterative process. In this process, performance data from past trials are analyzed using Gaussian processes, and the next hyperparameter combinations are selected probabilistically [58,59,60,61]. 

Bayesian optimization uses Gaussian processes to model the objective function f(θ). This method estimates a mean μ(θ) and a variance $\sigma^{2}(\theta )$ for each hyperparameter θ. A Gaussian process is defined as follows:

(9) $$f(\theta )∼GP(\mu (\theta ),k(\theta ,\theta^{\prime }))$$

Here, $k(\theta ,\;\theta^{\prime }),$ is the covariance function, which indicates the correlation of function values at two different hyperparameter points. In Bayesian optimization, the learning function a(θ) determines the next trial point by balancing exploration and exploitation strategies. The Expected Improvement (EI) function is often used:

(10) $$a(\theta )=E[max(f(\theta )-f(\theta^{+}),0)]$$

Here, $f(\theta^{+})$ represents the best available observation. The expected improvement function assesses the potential for improvement at a new point. Using Gaussian processes, the hyperparameter $\theta^{*}$ at which this acquisition function is maximized is found using Equation (11).

(11) $$\theta^{*}=arg\underset{\theta }{\max}a(\theta )$$

The model is trained with this new hyperparameter combination, its performance is measured, and the value of f(θ) is calculated. The Gaussian processes are updated with the new performance results, and the optimization process continues until the stopping criterion is reached. This iterative process provides a more accurate estimate of the objective function, making it possible to find the optimal hyperparameters with fewer trials [57,62].

This study aims to optimize the hyperparameters of the Weighted Boxes Fusion (WBF) algorithm such as Threshold, Skip Box Threshold, YOLO and RT-DETR weights using Bayesian optimization. The mathematical methods applied in the optimization process aim to improve the overall accuracy performance of the model by making the hyperparameter search both time and resource efficient.

### 2.6. Dataset

In this study, three different datasets consisting of panoramic radiographs are used for impacted tooth detection. The images in the datasets were carefully selected according to high resolution and quality standards and optimized for training the model. The dataset contains a total of 407 images, 304 images in PNG format with 540 × 380 pixels [44], 53 images in JPG format with 2041 × 1024 pixels [63] and 50 images in PNG format with 3100 × 1300 pixels [64]. This variety was specifically designed to provide results applicable to a larger population of teeth and to evaluate model performance at different resolutions.

**Figure 2:**
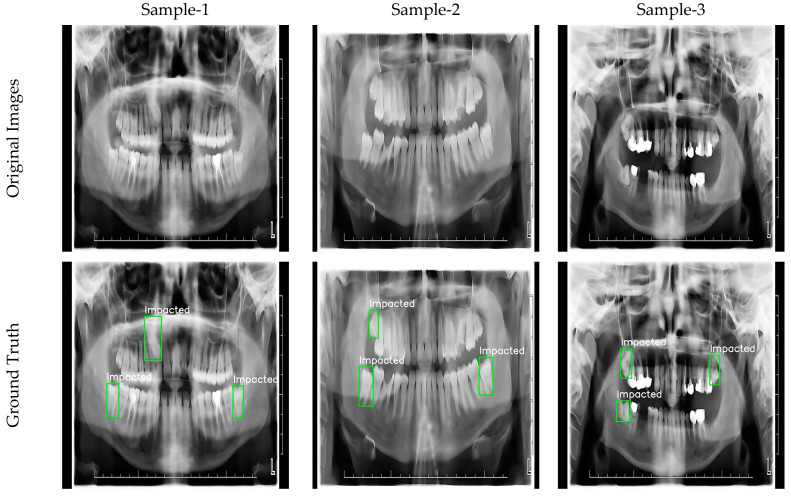
Sample labeled images.

Labeling procedures were carefully performed by experts in the field and all images were processed according to the YOLO format. The bounding box method was applied using the Roboflow platform and the positions of the impacted teeth were determined with high accuracy. This detailed labeling process provided a reliable basis for the training and testing phases of the model. Example images of the labeled dataset are shown in Figure 2.

This dataset has the potential to improve the overall performance of the model by providing samples at a wide range of resolutions for the detection of impacted teeth. The data selection and labeling process ensured a high degree of accuracy in the training and evaluation of the model, while allowing the results of the study to be generalized to a larger population of teeth. The resulting dataset is an important resource for impacted tooth detection studies, both in terms of its size and quality.

### 2.7. Implementation Details

The experimental studies were developed using the Python programming language and performed on a high-performance workstation with an Intel Core i9-14900K processor and NVIDIA RTX 4090 GPU, running on a Windows operating system. The dataset used was divided into three parts in order to provide an optimized process in the training, validation and testing phases of the model. In this context, 80% of the data (325 samples) are used for training, 5% (21 samples) for validation, and 15% (61 samples) for testing. The data split was performed only once, and the same datasets were used in all experimental studies to ensure consistency of analyses.

In the training process of the models in the experimental studies, default parameters optimized according to the YOLO and RT-DETR architectures were used. During training, the learning rate was set to 0.01, the momentum to 0.9, the weight decay to 0.0005, the batch size to 16, and the number of epochs to 500. The image size was fixed at 640 × 640 pixels. These hyperparameters were carefully chosen to efficiently train the model and improve its overall performance. To minimize the risk of overfitting during the training process, the EarlyStopping method was applied. This method automatically stops training if no improvement in the validation loss is observed for a certain period of time, preventing the model from overlearning unnecessarily. This optimized the training process of the model, resulting in more reliable and consistent results. This approach allowed the model to perform well on both training and test data.

The Weighted Boxes Fusion (WBF) algorithm was used to fuse the outputs of the YOLO and RT-DETR models. The parameters of the WBF algorithm were optimized using Bayesian optimization. In this process, hyperparameters such as *IoU* threshold, skip box threshold, YOLO weight and RT-DETR weight were optimized. The search ranges of the hyperparameters used in the optimization are shown in Table 1.

These optimized hyperparameters allowed the WBF algorithm to more effectively combine the strengths of the models and improve the overall detection accuracy. This approach, which is one of the highlights of this study, is one of the innovative methods used to improve model performance.

### 2.8. Performance Metrics

In this study, the performance of object recognition models is evaluated using key metrics such as precision, recall and *mAP50*. These metrics provide a comprehensive analysis of the prediction accuracy and reliability of the model. The performance metrics are defined mathematically in Equations (12)–(16):

(12) $$Precision=\frac{TP}{TP+FP}$$

(13) $$Recall=\frac{TP}{TP+FN}$$

Here, *TP*, *FP* and *FN* refer to true positive predictions, false positive predictions and false negative predictions respectively. The F1 score is a performance metric that represents the harmonic mean of precision and recall, offering a balanced measure of a model’s accuracy. It is particularly useful when dealing with imbalanced datasets, as it accounts for both false positives and false negatives. A higher F1 score indicates better overall performance in correctly identifying positive cases while minimizing errors. On the other hand, *IoU* measures the overlap between a predicted bounding box and the actual bounding box:

(14) $$IoU=\frac{Area\;of\;Overlap}{Area\;of\;Union}$$

where the estimate is considered a true positive if the *IoU* threshold is 0.5 or greater, and this is used in the *mAP50* calculations. The average precision (*AP*) is finding the approximation of the area under the PR curve. The actual area under the curve, where p(r) is the precision at recall r, can be defined as follows:

(15) $$AP=\int_{0}^{1}p(r)d_{r}$$

*mAP50* (mean average precision at *IoU* 0.5) represents the average of the calculated *AP* values for all classes:

(16) $$mAP50=\frac{1}{C}\sum_{c=1}^{C}{AP}_{c}$$

where C is the number of classes and ${AP}_{c}$ is the average precision for class *c* at the *IoU* threshold of 0.5. These metrics reveal the overall performance of the model by assessing its correct and incorrect predictions. They also provide a comprehensive framework for analyzing the accuracy, coverage and consistency of the predictions.

## 3. Experimental Results and Analysis

The experiments were conducted in three main phases: evaluation of individual models, evaluation of ensemble models, and evaluation of the optimized ensemble approach. The performance results based on these phases are detailed in the following subsections.

### 3.1. Individual Results

In this experimental study, different versions of the YOLOv8 and RT-DETR architectures are independently trained and tested. The performance of the models is evaluated both on normal images and on images enhanced with GAN-based super-resolution techniques. The performance results are presented in Table 2.

The results obtained from the analysis (as shown in Table 2) are as follows:

- The YOLOv8L model achieved the highest F1 values (94.6% and 94.5%) with both normal and GAN-based super-resolution images. It was also observed that mAP@0.5 (96.5%) improved with GAN-based images. These results show that YOLOv8L performs well with both image types. On the other hand, the YOLOv8X model performed similarly on normal and GAN-based images (88.8% mAP@0.5 and 93.7% F1). This suggests that the effect of GAN-based super-resolution on this model is limited.
- RT-DETR-L achieved the highest *IoU* score of 95.2% mAP@0.5 for normal images. However, this value decreased slightly to 94.7% for GAN-based images. In contrast, the GAN enhancement resulted in a significant increase in the F1 score (from 88.2% to 92.3%). On the other hand, RT-DETR-X showed a balanced performance on both normal and GAN-based images. However, there was a slight decrease in mAP@0.5 (92.3%) and F1 (91.7%) after GAN enhancement.

These results suggest that the impact of GAN-based super-resolution techniques on model performance may differ between model architectures. While YOLOv8L gives the best overall results for both mAP@0.5 and F1 metrics, the RT-DETR-L model shows a significant improvement after GAN application. This shows that the optimization of model components and the correct application of GAN-based enhancement techniques play a critical role in improving performance.

### 3.2. Ensemble Results

The second experimental study focused on the fusion of the outputs of the YOLO and RT-DETR architectures using the Weighted Boxes Fusion (WBF) algorithm. At this stage, the WBF algorithm was not subjected to any optimization process, and the fusion process was performed with default WBF parameters. The ensemble approach aims to achieve higher accuracy rates by combining the strengths of both models. In this study, different combinations of YOLOv8 and RT-DETR models were tested on both normal and GAN-enhanced images. The performance results are detailed in Table 3.

The results obtained from the analysis (as shown in Table 3) are as follows:

- In tests on normal images, the model combinations YOLOv8L + RT-DETR-X and YOLOv8X + RT-DETR-X achieved the highest F1 values (94.9% and 94.8%) and precision values (93.0% and 94.4%). These results indicate that the RT-DETR-X component provides high precision in normal images. On the other hand, the YOLOv8X + RT-DETR-L combination achieved the highest *IoU* value (95.6%) on normal images, but the recall rate (96.0%) remained similar to the other models.
- For GAN-enhanced images, the YOLOv8L + RT-DETR-X and YOLOv8L + RT-DETR-L combinations achieved the highest *IoU* and F1 scores (97.5% and 93.5%, 97.4% and 92.3%, respectively). YOLOv8L + RT-DETR-X performed particularly well in terms of precision (89.1%) and recall (98.4%). The combination YOLOv8X + RT-DETR-X showed a balanced performance on both normal and GAN-enhanced images, but the performance improvement after GAN enhancement was limited compared to the other combinations.

These results suggest that the ensemble strategy is effective in improving performance by combining the strengths of different models. In particular, GAN-based visualization was found to provide a significant improvement in *IoU* and recall rates in the YOLOv8L + RT-DETR-X combination. These results show that the proposed ensemble approach can make a significant contribution to the reliability and accuracy of clinical decision support systems. The detail enhancement effect of GAN-enhanced images is an important method to achieve better results in dental radiography.

### 3.3. Optimized Results

In this study, the parameters of the Weighted Boxes Fusion (WBF) algorithm are optimized using Bayesian Optimization to improve model performance. Bayesian optimization is a powerful method that makes the parameter search process more efficient by allowing an effective selection of hyperparameters. The optimization process focused on improving the precision, recall and F1 metrics. This process played a crucial role in improving the performance of the ensemble model. As a result of the optimization, models with mAP@0.5 values of 97% and above were considered and detailed performance results were obtained. The performance results of the optimized models are shown in Table 4.

The results obtained from the analysis (as shown in Table 4) are as follows:

1. YOLOv8L + RT-DETR-L: This model showed the highest accuracy of 98.3% with mAP@0.5. This means that the overall recognition accuracy of the model is quite high. The recall rate reached a remarkable 99.2%, indicating that the model was able to correctly detect almost all target objects. However, the precision remained relatively low compared to the other metrics at 86.7%. This suggests that the model’s false positive prediction rate could be improved. The F1 score reached 92.5% in the balance of precision and recall. The optimized parameters of the WBF algorithm for this model were determined as follows:
  - *IoU* Threshold (iou_thr): 0.3466;
  - Skip Box Threshold (skip_box_thr): 0.0340;
  - YOLOv8L Weight (weight1): 4.2111;
  - RT-DETR-L Weight (weight2): 2.9897.
2. YOLOv8L + RT-DETR-X: This model achieved a high accuracy of 97.5% mAP@0.5 and a remarkable performance with an F1 score of 96.0%. The precision was 93.8% and the recall 98.4%. This shows that the model provides a balanced and reliable performance. The optimized parameters of the WBF algorithm for this model were determined as follows:
  - *IoU* Threshold (iou_thr): 0.3597;
  - Skip Box Threshold (skip_box_thr): 0.0843;
  - YOLOv8L Weight (weight1): 7.7882;
  - RT-DETR-L Weight (weight2): 7.6551.

These results obtained with Bayesian optimization show that the WBF algorithm significantly improves its performance and that the correct choice of hyperparameters plays a critical role in the success of the models. The YOLOv8L + RT-DETR-L model showed strong performance in comprehensive object detection with high mAP and recall rates. However, the YOLOv8L + RT-DETR-X model offered a superior result in terms of overall accuracy and precision due to its balanced precision and recall ratios.

These results suggest that parameter refinement using Bayesian optimization is an effective tool to improve model performance, and that the WBF algorithm can achieve higher accuracy rates with optimized parameters.

### 3.4. Visualization

**Figure 3:**
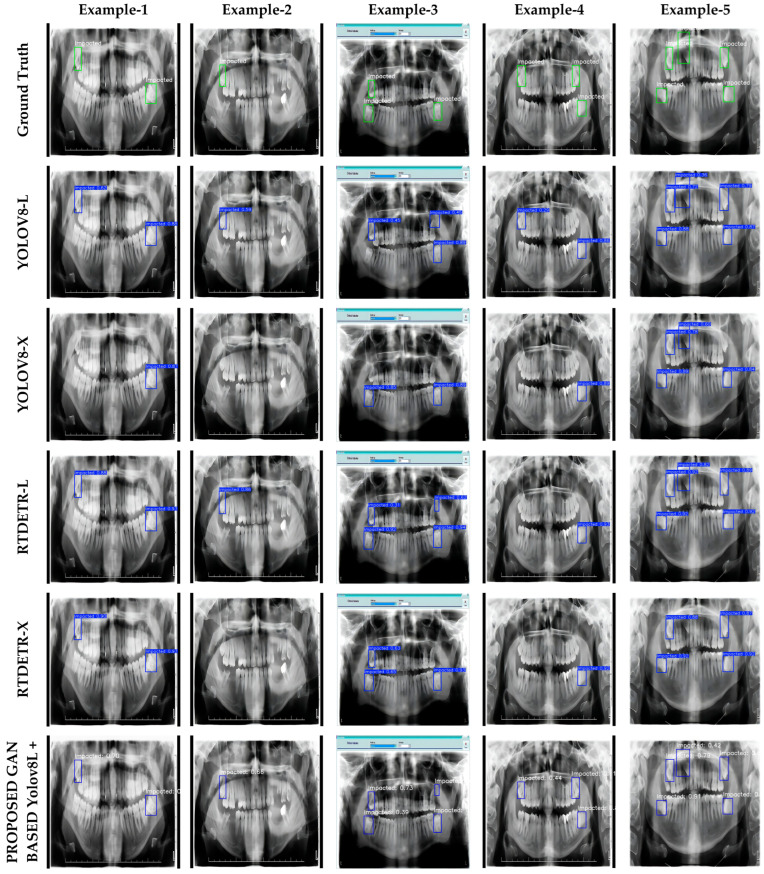

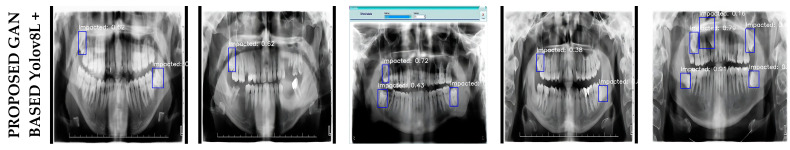
Visual estimation results obtained from experimental studies.

In this study, the visual results of YOLOv8-L, YOLOv8-X, RT-DETR-L, RT-DETR-X, and the proposed GAN-based optimization-assisted ensemble model are presented in detail in Figure 3. Figure 3 contains a series of panoramic images that visually compare the performance of the models used in the impacted tooth detection task. Each column represents a specific sample panoramic image, while each row shows the predictions of different model combinations. This structure allows a visual evaluation of the detection accuracy of the models. The first row contains the actual detections (ground truth), marked with green boxes, which are used as a reference to assess the accuracy of other models. Between the second and fifth rows, the predictions of the YOLOv8-L, YOLOv8-X, RT-DETR-L, and RT-DETR-X models are shown in blue boxes. The results in these rows show how the base models perform in different situations. The last row shows the predictions of the GAN-assisted RT-DETR-L and the proposed GAN-based optimization-assisted ensemble models. These results are included to highlight the effectiveness of the proposed method compared to other models. The visualizations provide a visual verification of the numerical results in Table 4 and clearly show the performance differences of the models.

The visualizations in Figure 3 clearly show how the embedded tooth detection performance of the proposed models responds to different scenarios. In particular, the optimized super-resolution GAN-based YOLOv8L + RT-DETR-L model showed an overall superior accuracy. The model correctly detected the vast majority of impacted teeth in all cases; however, in case 3, it incorrectly classified a non-impacted region as impacted. While this does not detract from the overall success of the model, it does suggest that further improvements are needed to reduce false positive predictions. In addition, the GAN-based YOLOv8L + RT-DETR-X model showed balanced performance, but was only able to correctly detect two of the three impacted teeth in Sample-4. This suggests that the model may occasionally have difficulty detecting impacted teeth in low-contrast regions or small sizes. However, considering the overall accuracy and precision of the model, it can be said that it provides a highly effective alternative for detecting impacted teeth.

On the other hand, the individual models YOLOv8-L, YOLOv8-X, RT-DETR-L and RT-DETR-X generally lagged behind the proposed GAN-based models. These models were found to under-detect, mis-locate or make false positive predictions in many cases. For example, these models failed to accurately localize target objects, particularly in low contrast areas or in complex panoramic images where impacted teeth overlap with other teeth. It was also found that in some cases, non-impacted teeth were incorrectly identified as impacted due to the model’s limited contextual understanding. This highlights the potential limitations of individual models in deep learning-based detection tasks and emphasizes the need for more advanced algorithms in complex image scenarios. Nevertheless, the fast processing capacity of individual models and their accuracy in certain situations suggest that they may be useful in lower cost and specific applications. However, in terms of overall performance, the integration of these models with the proposed GAN-based approaches provides significant improvements in terms of accuracy and reliability.

In conclusion, the visualizations presented in Figure 3 show that the proposed GAN-based models provide a significant advantage in terms of overall accuracy, precision and reliability compared to the individual models. However, addressing and improving the limitations observed in these models will contribute to obtaining more comprehensive and reliable results in clinical applications such as impacted tooth detection.

## 4. Discussion

### 4.1. Comparison of Previous Studies with Proposed Model

Studies on impacted tooth detection are limited in the literature and are generally based on pixel-based segmentation methods or regional detection approaches. Segmentation-based studies have generally achieved accuracies of 85% and higher [4,5,42,43,44]. However, regional detection methods have achieved higher accuracy and sensitivity rates, and the potential of these methods has been confirmed by studies using different deep learning models. The datasets, models used, and performance results of some important studies in the literature are summarized in Table 5.

In the studies presented in Table 5, although it is difficult to directly compare the performance of the models since they are based on different datasets, it is clearly seen that the proposed model shows a superior performance in terms of mAP and F1 score. According to the results obtained in this study, it is observed that the proposed model has high accuracy rates. It provides a significant performance improvement compared to similar studies in the literature, which emphasizes the superiority of the model. Looking at the comparative results in the literature, the YOLOv3 model used in [45] stands out with a mAP value of 96%. However, the proposed GAN-assisted YOLOv8L + RT-DETR-L model provided higher accuracy and precision than the existing methods in the literature with 98.3% mAP and a 92.5% F1 score. In addition, the GAN-assisted YOLOv8L + RT-DETR-X model provides a balanced and reliable result with 97.5% mAP and a 96% F1 score. These results show that the proposed model has made significant progress in the field of impacted tooth detection and outperforms the existing methods in the literature.

The main advantage of the proposed model is that it provides higher accuracy and consistency thanks to the GAN-based super-resolution techniques and ensemble approach. These methods significantly improved the overall performance by reducing false positive and negative predictions while increasing the clarity of the detected regions. Other studies in the literature usually use only a single model or limited combinations of models. In contrast, the proposed model not only integrates GAN-assisted super-resolution methods, but also achieves superior performance by combining powerful models such as YOLO and RT-DETR. Moreover, the model fusion process with the WBF algorithm emphasizes the strengths of each model while minimizing its weaknesses, resulting in more reliable results. In conclusion, this study has presented an innovative modeling strategy that is different from the existing work on impacted tooth detection and has made a significant contribution to the literature. The proposed model is considered to have a wide application potential, not only for impacted tooth detection, but also for various dental detection and analysis problems

### 4.2. Limitations

**Figure 4:**
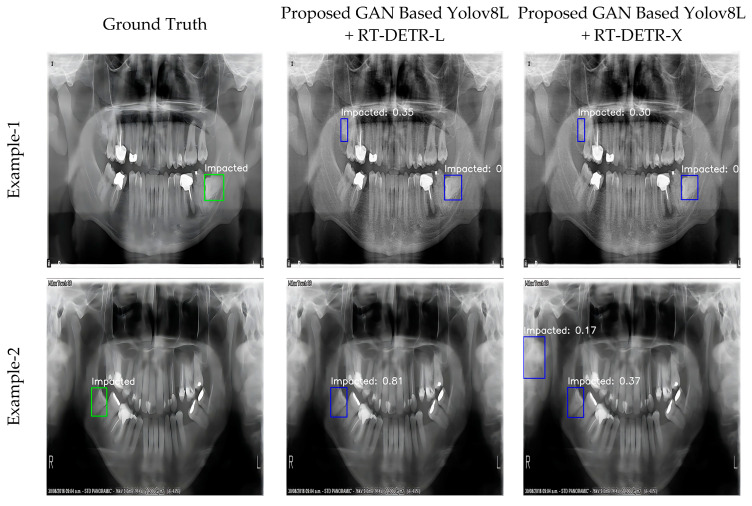
Sample images illustrating the limitations of the proposed model.

The proposed model is based on the integration of Convolutional Neural Networks (CNN) and transformer-based models on panoramic images enhanced with super-resolution techniques. The ability of the model to accurately detect the location of impacted teeth in all test images clearly demonstrates its overall success and reliability. However, it was observed that the model made errors in some cases, such as misidentifying non-impacted teeth as impacted teeth. Such limitations are critical to better understand the model’s performance in specific scenarios and to identify opportunities for future improvement. To better illustrate the limitations and challenges faced by the model, example images are shown in Figure 4.

The limitations of the model can be clearly seen in images Example-1 and Example-2 in Figure 4. In Example-1, the model incorrectly identified a fractured tooth as an impacted tooth, resulting in a false positive. Similarly, in Example-2, a region with no teeth was incorrectly labeled as an impacted tooth. These cases show that the model can misinterpret some complex structures and produce incorrect results in certain situations. These limitations provide important guidance by highlighting areas where the model needs to be improved. In particular, strategies such as larger and more diverse datasets, integration of more advanced feature extraction methods, and optimization of attention mechanisms could be considered in future studies. Furthermore, techniques to reduce false positive and false negative rates can be applied to make the model more robust in real-world applications.

In conclusion, this study highlights the strengths of the proposed model as well as its limitations, providing important insights that can guide future research. Optimizing the model to address these limitations will provide more effective and comprehensive solutions, not only for impacted tooth detection, but also for other dental detection and analysis problems.

### 4.3. Conclusions

This study presents an artificial intelligence-based solution for the detection of impacted teeth in panoramic radiographic images, combining YOLO and RT-DETR models with the Weighted Boxes Fusion (WBF) algorithm and Bayesian optimization. The proposed model achieved impressive results, including 98.3% mAP and a 96% F1 score, demonstrating its reliability and accuracy.

The model minimizes false positives and negatives while improving identification rates, making it a practical tool for clinical applications. It also shows potential for broader applications in dental and medical imaging tasks.

Future research could focus on validating the model on larger, diverse datasets and adapting it for other dental challenges, such as jaw anomalies, caries detection, or implant analysis.

## Figures and Tables

**Table 1:** The hyperparameters and search ranges.

Hyperparameters	Search Ranges
*IoU* threshold	0.1–0.9
Skip box threshold	0.001–0.1
Weight1 (YOLO weight)	1–10
Weight2 (RT-DETR weight)	1–10

**Table 2:** Normal and GAN-based individual performance results.

Models	Normal Images		GAN Images	
	mAP@0.5	F1	mAP@0.5	F1
Yolov8L	91.3	94.6	96.5	94.5
Yolov8X	88.8	93.7	88.8	93.7
RT-DETR-L	95.2	88.2	94.7	92.3
RT-DETR-X	94.2	92.2	92.3	91.7

**Table 3:** Ensemble performance results with default WBF algorithm.

Input	Hybrid Models	mAP@0.5	F1	Precision	Recall
Normal Images	Yolov8L + RT-DETR-L	95.4	88.8	82.7	96
	Yolov8X + RT-DETR-L	95.6	89.5	83.9	96
	Yolov8L + RT-DETR-X	95.3	94.9	93	96.8
	Yolov8X + RT-DETR-X	94.6	94.8	94.4	95.2
GAN Images	Yolov8L + RT-DETR-L	97.4	92.3	86.6	98.4
	Yolov8X + RT-DETR-L	95.5	93	89.6	96.8
	Yolov8L + RT-DETR-X	97.5	93.5	89.1	98.4
	Yolov8X + RT-DETR-X	94.6	94.8	94.4	95.2

**Table 4:** Performance results of the optimized models.

Input	Hybrid Models	mAP@0.5	F1	Precision	Recall
GAN Images	Yolov8L + RT-DETR-L	98.3	92.5	86.7	99.2
	Yolov8L + RT-DETR-X	97.5	96	93.8	98.4

**Table 5:** Comparison of studies on impacted tooth detection.

Reference	Dataset	Models	Performance Results
[3]	1084 panoramic radiographs	Faster R-CNN Inception v2	F1 score: 86.25%
[44]	440 panoramic radiographs	AlexNet-Faster R-CNN	mAP: 86%
		VGG16-Faster R-CNN	mAP: 87%
		ResNet50-Faster R-CNN	mAP: 91%
		YOLO v3	mAP: 96%
Our model	407 panoramic radiographs	GAN based Yolov8L + RT-DETR-L	mAP: 98.3% F1 score: 92.5%
		GAN based Yolov8L + RT-DETR-X	mAP: 97.5% F1 score: 96%

## Data Availability

The dataset used in this study is available on request from the corresponding author. The data are not publicly available due to privacy concerns.
